# Outcomes of patients with tracheostomy discharged from ICU to Transitional Care Unit and general wards

**DOI:** 10.1186/cc14297

**Published:** 2015-03-16

**Authors:** J Rubio, JA Rubio, E Palma, R Sierra, F Carmona, F Fuentes

**Affiliations:** 1 Hospital Universitario Puerta del Mar, Cadiz, Spain; 2 Hospital Infanta Cristina, Badajoz, Spain

## Introduction

Patients with a tracheostomy (TQ) tube in place discharged from the ICU to a general ward (GW) are a fragile group and the TQ may be a risk factor for morbidity and mortality [1,2]. For this reason they need closer monitoring and more airway care. The Transitional Care Unit (TCU) assists patients with serious medical conditions and bridges the gap between the ICU and home or long-term care facilities providing the necessary medical, nursing, psychological and rehabilitative care. The purpose of this study was to evaluate the impact of the TCU on outcomes of tracheostomized patients discharged from the ICU.

## Methods

We retrospectively reviewed medical records of ICU adults patients who had TQ when discharged to a new TCU and to a GW. The study was carried out in two tertiary care university hospitals from January 2007 to November 2014. Study variables were age, sex, APACHE II score, principal diagnosis, associated major procedures, length of stay in ICU and out in hospital, TCU and GW, Sabadell score, in-hospital mortality, types of tracheotomy procedure, decision to decannulate and discharge to home or long-care facilities.

## Results

In total 12,839 records of patients discharged from the ICU were analyzed. Tracheostomy was present in 133 patients. Two groups were defined: (1) TCU (*n *= 56) and (2) GW (*n *= 77). Patients of the TCU group were older (60.1 ± 13.1 vs. 54.9 ± 15.8 years; *P *< 0.05) with higher APACHE II score (23 (CI: 21.5 to 25.6) vs. 18.5 (CI: 17.1 to 19.9); *P *< 0.001), and had longer stay in the ICU (45.8 (CI: 38.2 to 53.3) vs. 28.4 (CI: 24.2 to 32.6) days; *P *< 0.001) and on the ward (71.1 (CI: 57.4 to 84.8) vs. 46.1 (CI: 33.7 to 58.2) days; *P *< 0.001) than those of the GW group. The GW group had category 1 of Sabadell score more frequently than the TCU group (25.9% vs. 8.9%; *P *= 0.019). Rates of nosocomial infections were similar in both groups. No significant differences on vasoactive use (50% vs. 40.2%), renal failure (23.2% vs. 20.7%), blood transfusions (25% vs. 23.2%), parenteral nutrition (10.7% vs. 12.9%), in-hospital deaths (14.3% vs. 24.6%), decannulation (55% vs. 50%), or discharge to home (53.6% vs. 37.7%) were found between groups 1 and 2, respectively.

## Conclusion

In our setting the TCU helps to care more safely for severe tracheostomized patients after ICU discharge, and furthermore facilitates discharge home.
